# Arum Palaestinum with isovanillin, linolenic acid and β-sitosterol inhibits prostate cancer spheroids and reduces the growth rate of prostate tumors in mice

**DOI:** 10.1186/s12906-015-0774-5

**Published:** 2015-08-05

**Authors:** Caitlin Cole, Thomas Burgoyne, Annie Lee, Lisa Stehno-Bittel, Gene Zaid

**Affiliations:** Likarda, LLC, 2002 W. 39th Ave, Kansas City, KS 66103 USA; Genzada Pharmaceuticals, LLC, 205 S. Broadway, Sterling, KS 67579 USA; Rockhurst University, 1100 Rockhurst Rd, Kansas City, MO 64110 USA; University of Kansas Medical Center, MS 2002, 3901 Rainbow Blvd., Kansas City, KS 66160 USA

**Keywords:** Prostate cancer, Arum palaestinum, Mice, Toxicity, Rat, Dose/response, Apoptosis, Cell culture

## Abstract

**Background:**

*Arum palaestinum* is a plant commonly found in the Middle East that is ingested as an herbal remedy to fight cancer. However, no studies have examined the direct effect of the plant/plant extract on tumor growth in an animal model.

**Methods:**

Verified prostate cancer cells were plated as 3D spheroids to determine the effect of extract from boiled *Arum Palaestinum* Boiss roots. In addition, male NU/NU mice (8 weeks old) with xenograft tumors derived from the prostate cancer cell line were treated daily with 1000 mg/kg body weight gavage of the suspension GZ17. The tumor growth was measured repeatedly with calipers and the excised tumors were weighed at the termination of the 3 week study. Control mice (10 mice in each group) received vehicle in the same manner and volume.

**Results:**

The number of live prostate cancer cells declined in a dose/dependent manner with a 24 h exposure to the extract at doses of 0.015 to 6.25 mg/mL. A fortified version of the extract (referred to as GZ17) that contained higher levels of isovanillin, linolenic acid and β-sitosterol had a stronger effect on the cell death rate, shifting the percentage of dead cells from 30 % to 55 % at the highest dose while the vehicle control had no effect on cell numbers. When GZ17 was applied to non-cancer tissue, in this case, human islets, there was no cell death at doses that were toxic to treated cancer cells. Preliminary toxicity studies were conducted on rats using an up-down design, with no signs of toxic effect at the highest dose. NU/NU mice with xenograft prostate tumors treated with GZ17 had a dramatic inhibition of tumor progression, while tumors in the control group grew steadily through the 3 weeks. The rate of tumor volume increase was 73 mm^3^/day for the vehicle group and 24 mm^3^/day for the GZ17 treated mice. While there was a trend towards lower excised tumor weight at study termination in the GZ17 treatment group, there was no statistical difference.

**Conclusions:**

Fortified *Arum palaestinum* Boiss caused a reduction in live cells within prostate cancer spheroids and blocked tumor growth in xenografted prostate tumors in mice without signs of toxicity.

## Background

The use of herbal remedies is widespread in both developing and developed countries [1]. This type of treatment is vitally important to populations without other healthcare options. In 2002, the World Health Organization estimated that 80 % of the world’s population in developing countries depended on plants and traditional medicine practitioners to meet their primary health care needs [2]. In addition, the use of herbals and the subsequent analysis of active ingredients has been important in the formation of many modern drugs.

Wild edible plants have always been an important source of therapeutics in traditional folk medicine. One of the most common plants ingested in the Palestinian region is the *Arum palaestinum* Boiss [3], also known as the Black Calla Lily. Often it is boiled and then the leaves are fried in olive oil and eaten because it is believed to protect from colon cancer. It is also commonly ingested as a tea. In fact, *Arum palaestinum* Boiss is one of the most commonly utilized plants for cancer patients in the region [3]. Nearly 40 % of the cancer patients ingesting the plant revealed that they anticipated a cure due to the plant. Yet, little is known about its active ingredients or its efficacy as an anticancer agent. In fact, there is a paucity of published articles devoted to the potential medicinal effects of this plant.

*Arum palaestinum* Boiss is a member of the Araceae family of plants, many of which contain polyphenols, alkaloids, flavone C-glycosides, flavonols, flavones, proantrhocyanidins and polyhydroxy alkalokds [4–7]. The plant is known to contain antioxidant activity [4, 8]. Some active compounds have been isolated directly from *Arum palaestinum* Boiss including two flavone C-glucosides (isoorientin and vitexin) [9]. Previously, we undertook studies to identify the active ingredients of *Arum palaestinum* Boiss and identified isovanillin, linolenic acid and β-sitosterol as major contributors. The purpose of this study was to determine whether native *Arum palaestinium* Boiss and its fortified version had anti-cancer activity against aggressive androgen-independent prostate cancer models *in vitro* and *in vivo*.

## Methods

### Plant extract preparation

Arum palaestinum Boiss roots and leaves were collected from wild samples or were cultivated on site. Samples were submitted to the Missouri Botanical Garden, St. Louis, MO for verification (voucher number, Croat 95,466(MO). Approximately 14 g of *Arum palaestinum* Boiss leaves and roots were placed in 1 L of water and brought to a boil for 15 min, followed by reduced heat for another 15 min. The mixture was filtered to remove large particles, resulting in the plant extract that was used in testing.

When working with plant extracts, the exact amount of each chemical component is difficult to control from plant to plant. Chemical components of the extract have been described elsewhere [10]. Three of the chemical components were fortified to known quantities, resulting in a compound termed “GZ17”. Isovanillin, linolenic acid and β-sitosterol (25 g each) (Sigma-Aldrich, St. Louis, MO) were diluted in the *Arum palaestinum* Boiss elixir composed of 15 g of *Arum palaestinum* Boiss extract in 3.79 L of H_2_O for a final concentration of 43.3 μM isovanillin, 23.8 μM linolenic acid, and 15.9 μM β-sitosterol. GZ17 was diluted in purified water and sonicated to achieve a 50 mg/mL stock solution and diluted in water to specified concentrations. The suspension was stored at 4 °C, shielded from light, and vortexed vigorously prior to use.

To concentrate the elixir for *in vitro* studies, a powder version was obtained by drying using rotoevaporation, followed by exposure to nitrogen to drive all remaining liquid out of the product, followed by manual pulverization.

### Cell culture and dose response

Vascular smooth muscle cells (PCS-100-021), dermal fibroblasts (HGF-1) and prostate cancer cells (22Rv1) were obtained from ATCC and verified within 2 passages of the use of the cells. Prostate cancer cells (22Rv1) are a recognized cell model of androgen-independent prostate cancer [11]. The cells were grown in RPMI supplemented with 10 % FBS + 1 % anti-anti. Cells were plated in a 3D cell culture system (Micromold, Likarda, LLC) to form spheroids of an average of 40 microns in diameter in 48 h. Methods for fabrication of the micromold and loading cultured cells into the micromold plate have been published previously [12, 13]. On day two, the spheroids are transferred to 96 well plates with an average of 50 spheroids/well.

Prostate cancer spheroids were exposed to increasing doses of the extract of *Arum palaestinium* Boiss, GZ17 (dose range from 0 – 6.25 mg/mL), or equivalent vehicle for 24 h prior to assays. Each trial was run with at least four replicates at each dose. At the completion of the exposure time, PrestoBlue (Life Technologies, Inc) was added to each well and fluorescence read (ex. 485/em. 560) on a microplate reader (Enspire Multimode, PerkinElmer) 4 to 6 h later. Results were averaged following background subtraction and normalized to untreated cells.

Toxicity studies were conducted on freshly isolated human islets, cultured vascular smooth muscle cells, and cultured fibroblasts. Fibroblasts were cultured in DMEM with 10 % FBS and 1 % strep/pen to 80 % confluency prior to testing. Vascular smooth muscle cells were grown in Vascular Cell Basal Medium supplemented with Vascular Smooth Muscle Growth Kit components (ATCC) to a confluency of 80–90 %.

Human islets were obtained from the Integrated Islet Distribution Program (City of Hope Hospital, Duarte, CA). Human islets were manually dispensed into a 96 well plate and exposed to increasing doses of GZ17 (4 replicates each) for 24 h. Followed by the addition of PrestoBlue (Life Technologies, Inc) to each well, fluorescence was read on a microplate reader (Enspire Multimode, PerkinElmer) 4 to 6 h later. Results were averaged following background subtraction.

### Mechanism of action

Cultured prostate cancer cells (22RVI) were seeded into 96-well plates at a density of 5,000 cells/well and allowed to grow for 48 h until 80–90 % confluent. The cells were exposed to 8 to 10 doses of GZ17 diluted in RPMI with 10 % FBS. Four replicates at each concentration were tested along with replicates of media only, and cells plus media only, 100 microliters total volume per well. Cells were exposed to GZ17 concentrations for 24 h at 37 °C and 5 % CO_2_. Caspase 6 Glo Reagent (Promega) was allowed to equilibrate to room temperature, and subsequently added in a 1:1 ratio to each well and left to incubate for 45 to 60 min. Luminescence was read with a plate reader (Perkin Elmer, Enspire). Changes in signal for assays were background subtracted and normalized to the 0 drug baseline.

### *In Vivo* toxicity

A single dose of 5000 mg/kg was administered by oral gavage to 10 week old female Sprague–Dawley rats, weighing between 170–200 gm (Ace Animals, Boyertown, PA). The animals were housed in an Association for the Assessment and Accreditation of Laboratory Animal Care (AAALAC)-accredited facility and the protocol was approved by the Institutional Animal Care and Use Committee (IACUC) of Eurofins Scientific following GLP standards. Over the subsequent 14 days, animals were weighed and activity was observed. Specifically, daily evaluations included skin, fur, eyes, mucous membranes, respiratory rate, and central nervous system behavior. Particular attention was directed for signs of tremors, convulsions, salivation, diarrhea and coma. At completion of the 14 days, the animals were euthanized, and the tissues and organs of the thoracic and abdominal cavities were examined.

### *In Vivo* efficacy

Male NU/NU mice (8 weeks old) were obtained from Charles River Laboratories, Inc. The animals were allowed to acclimate for a week and were housed in an Association for the Assessment and Accreditation of Laboratory Animal Care (AAALAC)-accredited facility and the protocol was approved by the Institutional Animal Care and Use Committee (IACUC) of Xenometrics, LLC. The mice and rats were held in cages with twelve hour light /dark periods, and were treated ethically throughout the study. Animal distress was monitored daily and no animals showed signs of distress throughout the study. Mice had access to food and water *ad libitum*, except for the 3 h period in the morning prior to treatment. Cancer cells from a prostate tumor cell line (PC3-MM2) were brought up in phosphate buffered saline (PBS). The immunocompromised mouse model using the androgen independent PC3-MM2 cells has been shown to be one of the most robust models for prostate cancer [14]. Approximately 10^6^ cells/100-200 μL of PBS were injected subcutaneously into the flank of each mouse. Tumors were allowed to develop to a measurable size prior to the initiation of the treatment with GZ17. Seven days following the injection of the cancer cells, tumors were visible and baseline tumor measurements were performed. The tumor size was measured with calipers twice weekly.

### Animal treatment protocol

Twenty mice were divided into treatment and vehicle groups (10 each) according to tumor size so that both groups had animals with matched tumor burdens. After nine days of monitoring the animals, GZ17 or the vehicle (purified water) was administered via an oral gavage of 1000 mg/kg in the morning at a volume of 20 mL/kg for 21 days. Food was removed from the cage 3 h prior to each dosing and was returned 1 h after the dosing. Animals were monitored twice daily for signs of acute or chronic pain due to the tumor burden and were euthanized by CO_2_ asphyxiation along with cervical dislocation. Tumors and organs were dissected, cleaned of fat and connective tissue and weighed.

### Statistical analysis

Statistical analysis was completed comparing the vehicle-treated and GZ17-treated mice using a Student’s *t*-test, or Mann Whitney Rank Sums when normality was not achieved. Repeated measures analysis of variance (ANOVA) was used to compare tumor sizes within animals; *p* < 0.05.

## Results

### *In Vitro* prostate cancer spheroid effect

**Fig. 1:**
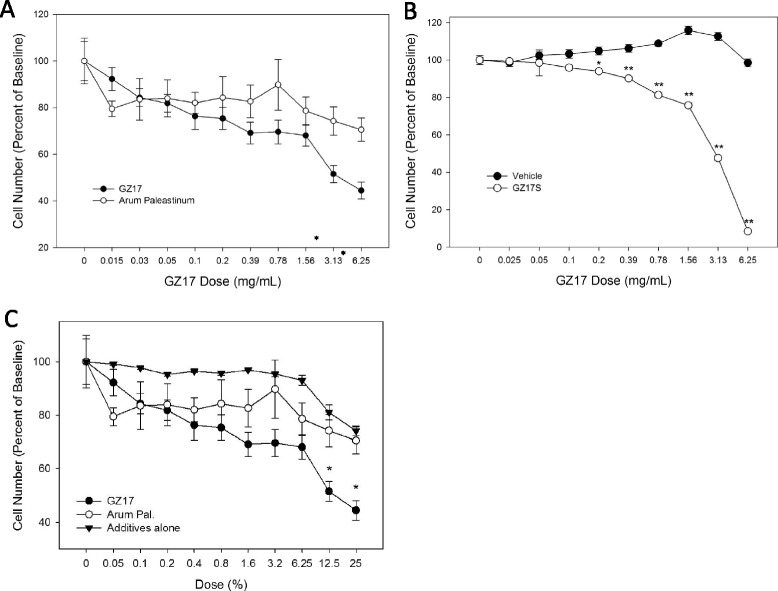
Prostate cancer spheroids respond to *Arum paleastinum* Boiss and the fortified GZ17. **a** Prostate cancer cells grown in 3D spheroids responded to *Arum palaestinum* Boiss and the fortified version, GZ17, in a dose dependent manner. GZ17 was more potent at inducing cell death compared to the plant extract with statistically lower cell numbers at 3.13 and 6.25 mg/ml. **b** Compared to the vehicle, which had no cell death effect, GZ17 was statistically more potent at inducing cell death. **c** The 3 fortifying components that when added to Arum palaestinum, create GZ17, were tested alone on prostate cells. The additives (open circles) had little effect on cell viability. *indicates *p* < 0.005; **indicates *p* < 0.001

Prostate cancer spheroids were exposed to increasing doses of *Arum palaestinum* Boiss extract or to the fortified version of the plant extract called GZ17. Both showed a dose-dependent increase in cell death at concentrations ranging from 0.015 to 6.25 mg/mL, but the GZ17 was slightly more potent at inducing cell death in the spheroids (Fig. 1a). IC50 values could not be calculated, as the maximal effect could not be reached with the highest concentration of the pure plant extract due to its precipitation at higher concentrations. For this reason, the extract was dried to obtain a solid form for reconstitution in water. The effect on prostate cells was more evident using the reconstituted version of GZ17 (Fig. 1b). It was compared to the vehicle control, which induced no cell death at the same concentrations. In contrast, the GZ17-treated plates lost 96 % of the cells at the highest GZ17 dose (6.25 mg/mL).

The fortifying components added to Arum palaestinum Boiss to create GZ17 were tested alone for any effect on prostate cancer cells. Figure 1c illustrates the lack of response when the 3 fortifying components (isovanillin, linolenic acid and β-sitosterol) were applied to the cancer cells alone. In contrast, when the 3 were combined with the plant to form GZ17, the cell death rate was greatly enhanced to the point that nearly all cells were dead at the highest tested dose.

**Fig. 2:**
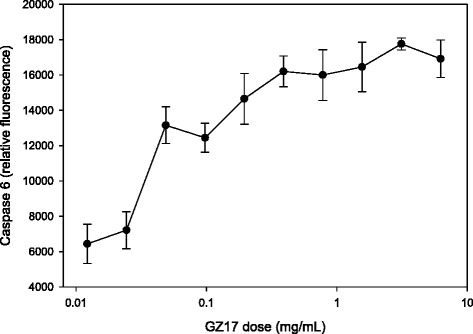
GZ17 induces a dramatic increase in caspase 6 levels. Prostate cancer spheroids were exposed to increasing doses of GZ17 and caspase 6 levels measured. Caspase 6 increased 3 times, beginning at low doses. * indicates significant increase over baseline, *p* < 0.005

To determine the method of cell death, activation of caspase proteins were surveyed. Figure 2 shows dose response (log dose) of caspase 6 in prostate cancer when exposed to GZ17. While cell death was measured at GZ17 doses of 0.2 mg/mL GZ17 (Fig. 1b), caspase activation was measured far earlier below 0.1 mg/mL.

**Fig. 3:**
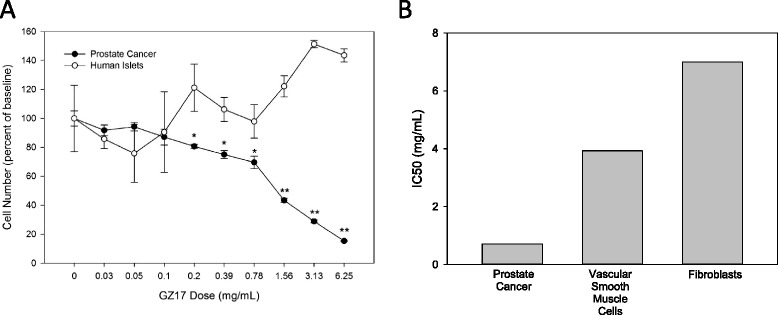
GZ17 is less toxic on non-cancer human islets. **a** The cell death inducing activity of GZ17 was compared in human prostate cancer spheroids and human non-cancerous islets. GZ17 failed to induce cell death in the human islets, but consistently caused a dramatic loss of cancer cells. *indicates *p* < 0.01 and ** indicates *p* < 0.001 when comparing the islet and prostate cancer results at each dose. **b** The IC50 for vascular smooth muscle cells and human fibroblast cells were compared to the value for prostate cancer cells

To determine whether the cell death effect of GZ17 was specific to cancer cells, non-cancerous human islets were exposed to the same doses of GZ17 for 24 h. GZ17 caused no cell-death on the human islets with cell numbers greatest at the highest doses (Fig. 3a). In contrast, GZ17 caused a dose-specific cell death in the prostate cancer spheroids with a half maximal inhibitory concentration (IC_50_) of approximately 3 mg/mL and only 8.5 % of the total number of cells alive when exposed to 6.25 mg/mL GZ17. Using the same methods, IC_50_ levels were determined for GZ17 on non-cancerous vascular smooth muscle cells and fibroblasts. Figure 3b summarizes the higher dose required to achieve the IC_50_ level in the fibroblasts and smooth muscle cells compared to the prostate cancer cells.

### *In Vivo* toxicity

An up and down toxicity design was used to gather preliminary data on *in vivo* toxicity of the ingested GZ17. A single female rat was given a one-time maximal dose of GZ17 (5000 mg/kg). The rat was observed for signs of gross toxicity, mortality and behavioral changes daily for the following 2 weeks. No signs of pain related behavior, respiratory or cardiovascular distress, tremors, convulsions, salivation, diarrhea, or coma were noted. Skin and fur, eye and mouth mucosa, and central nervous system function were normal. Upon necropsy at the completion of the study, all abdominal and thoracic tissues were normal. With no lethality or toxicity signs or symptoms noted, two additional rats were dosed at the same 5000 mg/kg level with similar outcomes. The subsequent animal subjects also showed no gross signs of toxicity and no organ abnormality at the time of necropsy. Table 1 provides the weight gain for each animal following the maximal dose, demonstrating normal weight gain.

**Table 1:** Rat toxicity

Rat number	Initial weight (gm)	Day 7 weight (gm)	Completion weight (gm)	% weight gain
1	170	193	239	71
2	200	219	248	81
3	176	194	240	73

### *In Vivo* prostate cancer effect

Having determined that GZ17 contained anti-cancer properties on prostate cancer cells *in vitro* with no signs of toxicity in rats, the compound was used for complimentary *in vivo* studies, employing a mouse model of prostate cancer. Prostate cells were injected into all mice to form tumor-like masses prior to treatment with GZ17. The animals used in this study appeared healthy throughout the treatment. Redness was noted over the tumor region 7 days after the injection of tumor cells but was resolved without obvious stress to the animal.

**Fig. 4:**
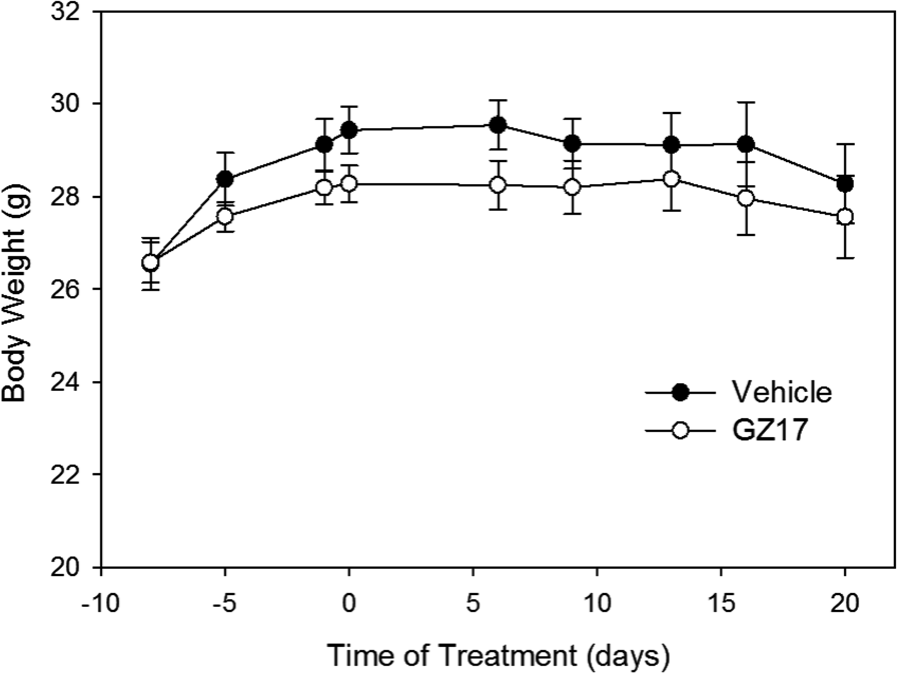
Animal weight. Mice were weighed prior to treatment with GZ17 or the vehicle. Treatment began on day 0. There was no statistical difference in the body weights of either group of mice throughout the 3-week study

Control mice received the vehicle, while the experimental group received the extract version of GZ17 via oral gavage once/day for 3 weeks. Mice were weighed twice weekly. There was no difference in body weight between the two groups throughout the study (Fig. 4). There was a 6.1 % increase in average weight of the vehicle-treated mice, and a 3.5 % increase in average weight of the GZ17-treated animals. The difference was not statistically significant and began prior to the initiation of the GZ17 treatment.

**Fig. 5:**
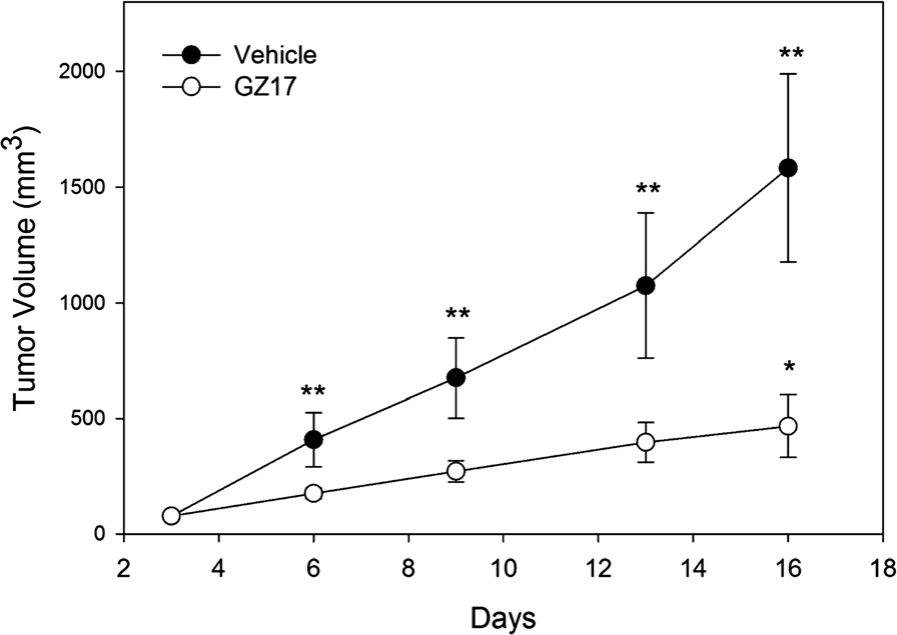
Tumor volume. Tumor volume was measured in 3 dimensions with calipers. The size of the tumors and the rate of tumor growth were both statistically greater in the vehicle-treated mice, compared to the GZ17. **indicates p< 0.001; *indicates p < 0.001 for comparisons between effects of days of treatment and baseline tumor volume within groups (repeated ANOVA)

Tumor sizes were measured twice per week. Prostate tumors grew steadily throughout the 3-week treatment period in the vehicle-treated mice. There was a 122 % increase in tumor size by the end of the study in the vehicle-treated animals (Fig. 5)

. GZ17 slowed tumor growth when compared to the vehicle group with only a 9.6 % increase in tumor size. By day 6 and for all subsequent measurements there was a statistically significant increase in the tumor volume of the vehicle-treated mice. There was no statistically significant increase in tumor size until day 16 in the GZ17-treated group. The rate of tumor volume increase was 73 mm^3^/day for the vehicle group and 24 mm^3^/day for the GZ17 treated mice. Tumor growth delay was measured when control tumors reached a volume of 500 mm^3^, which occurred on day 7.1 in the control animals and day 16.3 in the GZ17-treated mice. On the day of euthanasia, the average tumor volume was more than double in the controls compared to the treated mice. Furthermore, treated mice showed no changes in behavior, activity levels, grooming or other indications of altered general health. At completion of the study, the tumors were excised and weighed. The tumors in the vehicle-treated mice weighed 58 % more than the treated group, but there was no statistical difference between the two groups.

In order to assess whether GZ17 caused unintended side effects, organs from all animals in the 3-week treatment study were removed, inspected and weighed. In agreement with the earlier rat studies, there were no adverse signs of drug effect such as altered gross morphology of the organs analyzed, including indications of metastasis, in the brain, heart, kidneys, liver, lungs, or spleen. There were no differences in the weights of the six organ tissues between the vehicle and GZ17 treated groups, and when organ weights were normalized to the body weight of each animal (Table 2).

**Table 2:** Mouse organ weight

Tissue	Vehicle	GZ17	P
Brain	0.40 ± 0.03	0.40 ± 0.04	0.94
Heart	0.18 ± 0.01	0.19 ± 0.01	0.35
Kidney	0.44 ± 0.03	0.46 ± 0.02	0.59
Liver	1.41 ± 0.08	1.40 ± 0.10	0.86
Lung	0.22 ± 0.02	0.28 ± 0.03	0.16
Spleen	0.09 ± 0.01	0.09 ± 0.01	0.57

## Discussion

Complementary medicine is commonly used among cancer patients. In fact, 52 % of cancer patients taking chemotherapy admitted to the use of herbal teas, yet nearly half of these individuals had not shared this information with their oncologists [15]. Classical research focusing on a single active compound disregards the historical knowledge of the traditional remedy and the source of the plant. One of the main challenges in working with herbs and understanding their mechanism of action is the fact that plant-based compounds contain a number of potentially active chemicals and the quantity and quality of those compounds may differ based on the plant’s environment during the growing season [16]. For this reason, the work here identified three new components of A*rum palaestinium* Boiss. The fortification of the plant extract with these compounds ensured a threshold amount for each component regardless of differences inherent between plants. Alone, these three components failed to induce dramatic cell death, but when added to the plant extract a greater level of cell death was noted at lower doses.

The tumor-limiting effect of GZ17 in this study, in both cell culture and in mice, builds on previous publications showing *in vitro* inhibition of tumor growth by native A*rum palaestinum* Boiss. In previous studies, the ethyl acetate-identified fraction of the plant was shown to suppress *in vitro* cell proliferation of breast cancer (MCF07 cells) [4] and lymphoblastic leukemia cells (1301) in a dose-dependent manner [7]. However, the same fraction failed to suppress growth of a liver cancer cell line (Hep G2) [7]. This is the first published paper showing *in vitro* cancer cell suppression for prostate cancer with activation of caspase 6, as well as *in vivo* tumor suppression of prostate cancer in mice. Caspase 6 has been termed an effector caspase, along with caspases 3 and 7, which are activated intrinsically or extrinsically by one of the initiator caspases, 2, 9, 8, or 10 [17]. We confirm here that caspase 6 levels increase at doses lower than cell death was measured. Caspase 6 has been long identified as being associated with prostate cancer. In fact, out of 107 prostate human adenocarcinomas analyzed, 61 % of prostate tumors had high caspase 6 activity, suggesting apoptotic deregulation may occur in early stages of prostate cancer [18]. Further, resistance to chemotherapies has been associated with changes in the caspase genes, with caspase 6 in particular showing association to drug resistance [19]. Thus, caspase 6 has been established as an important marker for prostate cancer [18].

The *in vivo* studies illustrated the efficacy by inhibiting the growth rate of the xenotransplanted prostate tumors in mice. Perhaps one of the most important findings of this paper is that an extremely high dose of the extract (1000 mg/kg body weight) administered for 3 weeks did not cause obvious side effects in the mice or visible changes in any organs. These results are consistent with the *in vitro* toxicity study on non-cancerous human islets and the rat toxicity study at 5000 mg/kg, and provide surprising results as to how well the plant extract was tolerated in both rats and mice.

## Conclusions

While there was previously anecdotal evidence that *Arum palaestinum* Boiss benefits cancer patients [3], this is the first published study measuring the tumor suppressing effect of the fortified plant on an animal model of cancer. These results clearly demonstrate an effect of fortified *Arum palaestinum* Boiss on suppressing prostate cancer cells and prostate tumors in mice. While the results are encouraging, additional studies should be done to elucidate the underlying mechanism(s) of action and the potential activity on other types of cancers.

## Abbreviations

RPMI: Roswell Park Memorial Institute
FBS: Fetal bovine serum
AAALAC: Association for the Assessment and Accreditation of Laboratory Animal Care
PBS: Phosphate-buffered saline
CO_2_: Carbon dioxide
ANOVA: Analysis of variance
IC_50_: Half maximal inhibitory concentration

## References

[1] Olaku O, White J (2011). Herbal therapy use by cancer patients: a literature review on case reports. Eur J Cancer..

[2] The World Health Organization. Traditional medicine strategy 2001–2005. WHO Publications: Geneva. Accessed 2014; [http://whqlibdoc.who.int/hq/2002/WHO_EDM_TRM_2002.1.pdf?ua=1]

[3] Ali-Shtayeh M, Jamous RM (2008). Traditional knowledge of wild edible plants used in Palestine (Northern West Bank): A comparative study. J Ethnobiol Ethnomed..

[4] Husein A, Ali-Shtayeh MS, Jondi WJ, Zatar NA, Abu-Reiday IM, Jamous RM (2014). *In vitro* antioxidant and antitumor activities of six selected planted used in traditional Arabic Paestinian herbal medicine. Pharm. Biol..

[5] Kite G, Sharp HJ, Hill PS, Boyce PC (1997). Polyhydroxyalkaloids in the aroid tribes nephthytideae and aglaonemateae: Phytochemical support for an intertribal relationship. Biochm. Syst. Ecol..

[6] Williams C, Harbome J, Mayo S (1981). Anthocyanin pigments and leaf flavonoids in the family Araceae. Phytochemist..

[7] El-Desouky S, Kim KH, Ryu SY, Eweas AF, Gamai-Eldeen AM, Kim YK (2007). A new pyrrole alkaloid isolated from Arum palaestinum Boiss and its biological activities. Arch Pharm Res.

[8] Al-Mustafa A, Al-Thunibat O (2008). Antioxidant activity of some Jordanian medicinal plants used traditionally for treatment of diabetes. Pak J Biol Sci.

[9] Afifi F, Khalil E, Abdalla S (1999). Effect of isoorientin isolated from Arum palaestinum on uterine smooth muscle of rats and guinea pigs. J Ethnopharmacol.

[10] Zaid GH, Burgoyne, TW, Wolf BA. Methods and dosage forms for the treatment of human cancers. US Patent. 2011; US8039025 B1(US 13/157.249).

[11] Sramkoski M, Pretlow TG, Giaconia JM, Pretlow TP, Schwartz S, Sy M-S, Marenco SR, Rhim JS, Zhang D, Jacobberger JW (1999). A new human prostate carcinoma cell line, 22Rv1. In Vitro Cell Dev Biol.

[12] Ramachandran K, Peng X, Bokvist K, Stehno-Bittel L (2014). Assessment of reaggregated human pancreatic islets for secondary drug screening. Br J Pharmacol.

[13] Ramachandran K, Williams SJ, Huang HH, Novikova L, Stehno-Bittel L (2013). Engineering islets for improved performance by optimized reaggregation in a micromold. Tissue Eng Part A.

[14] Eskew JD, Sadikot T, Morales P, Duren A, Dunwiddie I, Swink M, Zhang X, Hembruff S, Donnelly A, Rajewski RA, Blagg BSJ, Manjarrez JR, Matts RL, Holzbeierlein JM, Vielhauer GA (2011). Development and characterization of a novel C-terminal inhibitor of Hsp90 in androgen dependent and independent prostate cancer cells. BMC Cancer..

[15] Pihlak R, Liivand R, Trelin O, Neissar H, Peterson I, Kivistik S, Lilo K, Jaal J (2014). Complementary medicine use among cancer patients receiving radiotherapy and chemotherapy: methods, sources of information and the need for counselling. Eur J Cancer Care.

[16] Poojari R, Patil A, Gota V (2012). Development of botanical principles for clinical use in cancer: where are we lacking?. J Postgrad Med.

[17] Shalini S, Dorstyn L, Dawar S, Kumar S (2015). Old, new and emerging functions of caspases. Cell Death and Differentiation..

[18] Yoo NJ, Kim MS, Park SW, Seo SI, Song SY, Lee JY, Lee SH (2010). Expression analysis of caspase-6, caspase-9 and BNIP3 in prostate cancer. Tumori.

[19] Reinhold WC, Kouros-Mehr H, Kohn KW, Maunakea AK, Lababidi S, Roschke A, Stover K, Alexander J, Pantazis P, Miller L, Liu E, Kirsch IR, Urasaki Y, Pommier Y, Weinstein JN (2003). Apoptotic susceptibility of cancer cells selected for camptothecin resistance: gene expression profiling, functional analysis, and molecular interaction mapping. Cancer Res.

