# Erratum to: Arum Palaestinum with isovanillin, linolenic acid and β-sitosterol inhibits prostate cancer spheroids and reduces the growth rate of prostate tumors in mice

**DOI:** 10.1186/s12906-015-0854-6

**Published:** 2015-09-14

**Authors:** Caitlin Cole, Thomas Burgoyne, Annie Lee, Lisa Stehno-Bittel, Gene Zaid

**Affiliations:** Likarda, LLC, 2002 W. 39th Ave, Kansas City, 66103 KS USA; Genzada Pharmaceuticals, LLC, 205 S. Broadway, Sterling, 67579 KS USA; Rockhurst University, 1100 Rockhurst Rd, Kansas City, 64110 MO USA; University of Kansas Medical Center, MS 2002, 3901 Rainbow Blvd., Kansas City, 66160 KS USA

Erratum

Unfortunately, the original version of this article [1] contained an error. The presentation of Fig five was incorrect due to the figure not appearing along with the information and the legend. This is an update and Erratum to the original article. The corrected Figure five can be seen below (Fig. 1):

**Fig. 1:**
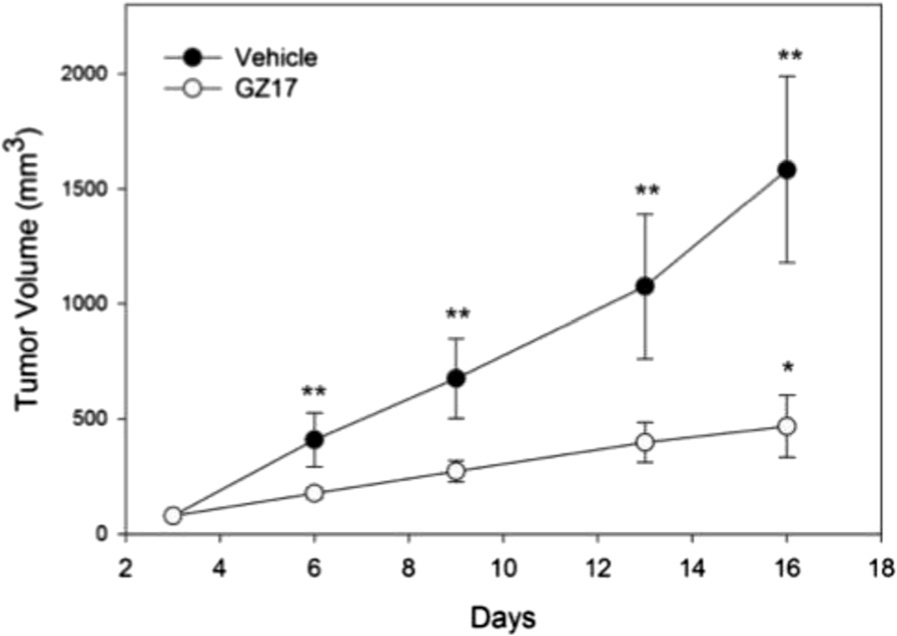
Tumor volume. Tumor volume was measured in 3 dimensions with calipers. The size of the tumors and the rate of tumor growth were both statistically greater in the vehicle-treated mice, compared to the GZ17. **indicates *p <* 0.001; *indicates *p <* 0.001 for comparisons between effects of days of treatment and baseline tumor volume within groups (repeated ANOVA)
